# Ribonucleotide Reductase Large Subunit M1 Predicts Poor Survival Due to Modulation of Proliferative and Invasive Ability of Gastric Cancer

**DOI:** 10.1371/journal.pone.0070191

**Published:** 2013-07-29

**Authors:** Qinchuan Wang, Xiyong Liu, Jichun Zhou, Yasheng Huang, Shengjie Zhang, Jianguo Shen, Sofia Loera, Xiaoming Yuan, Wenjun Chen, Mei Jin, Stephen Shibata, Yingbin Liu, Peiguo Chu, Linbo Wang, Yun Yen

**Affiliations:** 1 Department of Surgical Oncology, Affiliated Sir Runrun Shaw Hospital, Zhejiang University School of Medicine, Hangzhou, China; 2 Department of Pathology, Affiliated Sir Runrun Shaw Hospital, Zhejiang University School of Medicine, Hangzhou, China; 3 Department of Molecular Pharmacology, City of Hope Comprehensive Cancer Center, Duarte, California, United States of America; 4 Department of Anatomic Pathology, City of Hope Comprehensive Cancer Center, Duarte, California, United States of America; 5 Department of Medical Oncology, City of Hope Comprehensive Cancer Center, Duarte, California, United States of America; 6 Department of General Surgery, Shanghai Xinhua Hospital, Shanghai, China; 7 Taipei Medical University, Taipei, Taiwan; Sapporo Medical University, Japan

## Abstract

**Objectives:**

We aimed to investigate the prognostic value of RRM1 in GC patients.

**Methods:**

A total of assessable 389 GC patients with clinicopathological and survival information were enrolled from City of Hope (COH, n = 67) and Zhejiang University (ZJU, n = 322). RRM1 protein expression was determined by immunohistochemistry on FFPE tissue samples. Kaplan-Meier and Cox analyses were used to measure survival. Ras/Raf activity and invasion assays were used to evaluate the role of RRM1 in GC cell lines.

**Results:**

*In vitro* experiments demonstrated RRM1 activated Ras/Raf/MAPK signal transduction and promoted GC cell proliferation. Meanwhile, RRM1 expression was significantly associated with lymph node involvement, tumor size, Ki67 expression, histological subtype and histological grade in the GC tissue samples (*p*<0.05). Kaplan-Meier analysis illustrated that high RRM1 expression predicted poor survival in GC patients in the COH and ZJU cohorts (log-rank *p*<0.01). In multivariate Cox analysis, the hazard ratios of RRM1 for overall survival were 2.55 (95% CI 1.27–5.15) and 1.51 (95% CI 1.07–2.13) in the COH and ZJU sets, respectively. In particular, RRM1 specifically predicted the outcome of advanced GCs with poor differentiation and high proliferative ability. Furthermore, inhibition of RRM1 by siRNA significantly reduced the dNTP pool, Ras/Raf and MMP-9 activities and the levels of p-MEK, p-ERK and NF-κB, resulting in growth retardation and reduced invasion in AGS and NCI-N87 cells.

**Conclusions:**

RRM1 overexpression predicts poor survival in GC patients with advanced TNM stage. RRM1 could potentially serve as prognostic biomarker and therapeutic target for GCs.

## Introduction

Gastric adenocarcinoma (gastric cancer, GC) is the second leading cause of cancer-associated mortality worldwide, particularly in Asian and developing countries, where approximately one million cases are diagnosed annually [1]–[2]. The highest mortality (28.1 per 100,000 males, 13.0 per 100,000 females) from GC has been reported in East Asia, which includes China, Japan and Korea [2]. Conventional treatment modalities, including surgery, chemotherapy and radiotherapy, have shown a survival benefit for GC patients [3]–[6]. However, the 5-year survival rate is still disappointing, and most GC patients die of disease complications and relapse [3], [7]–[8]. Several biomarkers are being investigated with the aim of predicting survival and improving outcomes in patients with GC [9]. So far, few of the biomarkers are widely used clinically for GC.

Ribonucleotide reductase (RNR) is considered a therapeutic target for cancer treatment. RNR is a time-limited enzyme that catalyzes the conversion of ribonucleoside diphosphates to deoxyribonucleoside diphosphates through *de novo* metabolism of endogenous nucleotides [10]. RNR inhibitors, such as hydroxyurea, have been widely used to treat leukemia and solid tumors [11]–[13]. However, the application of RNR inhibitors has been limited due to low efficacy, drug resistance and side effects [14]. Most of the limitations of RNR inhibitors are caused by non-specific targeting. Human RNR consists of three subunits, the large subunit M1 (RRM1) and two small subunits M2 and M2B. Each small subunit can complex with RRM1 to form an active holoenzyme [10]. M2 level predicts poor prognosis in multiple cancers, including lung cancer, pancreatic cancer and GC [15]–[18]. M2B seems to play a role in suppressing malignancy and is associated with better survival in colorectal cancers, according to our previous research [19]–[20]. However, the biological roles of RRM1 are not identical among these cancers. We have investigated the mRNA levels of RRM1 in cancer and corresponding normal tissue sections using the ONCOMINE database (www.oncomine.com). Under selected conditions (*p*<0.05, fold change>2, gene rank = top 10%, all data types), the RRM1 mRNA was up-regulated in 39 unique analyses and down-regulated in 11 unique analyses. RRM1 mostly increased in the sections from bladder, cervical, colorectal, head and neck, liver and lung cancers, as well as melanoma and sarcoma. Meanwhile, RRM1 was down-regulated in breast cancer, leukemia and lymphoma. The mammalian RNR subunit R1 (similar to RRM1 in human) plays a role in malignancy suppression by inactivating the Ras/Raf/MAPK signaling pathway in Ras-transformed 3T3 cells (mice) [21]–[22]. Further outcome studies have demonstrated that high RRM1 expression (along with ERCC1 or PTEN up-regulation) is a determinant of optimal survival in early-stage non-small-cell lung cancer (NSCLC) [23]–[26]; however, other studies have shown seemingly conflicting results [27]–[29]. RRM1 overexpression is related to resistance to gemcitabine in NSCLC and pancreatic cancer, which results in a poor outcome [30]. These studies indicate that the role of RRM1 in cancer remains controversial, even in the same cancer type at different stages or under different therapies [31]–[32]. RRM1 has been suggested to have other biological roles besides forming RNR holoenzymes to convert NDP to dNDP, which could partially explain the low efficacy of RNR inhibitors in cancer treatment. Therefore, extensively investigating the roles of RRM1 would be helpful in developing novel RNR inhibitors for treating cancer specifically. GC is one of most common cancers in the world, but RNR inhibitors, such as hydroxyurea and gemcitabine, have not been used commonly because of low efficacy. On the other hand, the impact of RRM1 on GC outcome has never been examined. Therefore, it would be valuable to explore the biological role of RRM1 in GC cells and evaluate the clinical meaning of RRM1 overexpression in GC patients.

In this study, the roles of RRM1 in regulating cell proliferation and invasion through the Ras/Raf signaling pathway in GC cell lines were investigated. Meanwhile, RRM1 protein expression and the outcome of 67 GC patients from City of Hope National Medical Center (COH) were also determined. The findings were further validated in 322 GC patients from the Affiliated Sir Runrun Shaw Hospital of Zhejiang University (ZJU). Our findings suggest that RRM1 predicts poor survival in GCs and could potentially serve as a prognostic biomarker and therapeutic target in GC patients.

## Materials and Methods

### Ethic Statement

The protocol of this study was reviewed and approved by the institutional review board (IRB) of the City of Hope National Medical Center and Zhejiang University, respectively. Written informed consent was obtained by all the patients enrolled in this study. The eligible GC samples were collected from City of Hope National Medical Center (COH set) and the Affiliated Sir Runrun Shaw Hospital, Zhejiang University (ZJU set), respectively.

### Patients

The inclusion criteria for participants included the following: (i) gastric adenocarcinoma with a pathological diagnosis; (ii) informed consent or waiver of consent provided by the patient; and (iii) follow-up information available. We excluded GC patients with (i) failure to provide informed consent; (ii) non-adenocarcinoma or multiple cancers; (iii) no tissue sample obtained; (iv) loss of contact after surgery; or (v) stage IV GC without palliative surgery. The COH set included a series of 67 eligible GC patients who received surgery with R0(58 cases), R1(8 cases) or R2(1 case) resection in City of Hope National Medical Center from January 1989 to December 2006. The participants in the COH set consisted of 51 Whites, 2 African-Americans and 14 Asians. In the ZJU set, eligible 322 GC patients (242 cases with R0 resection, 67 cases with R1 resection and 13 cases with R2 resection) were enrolled. They were obtained their surgical operation during 1997 to 2001. All GC patients in the ZJU set were Asian (Chinese). In the ZJU set, 153 of 322 patients had post-surgery adjuvant chemotherapy. The combination chemotherapy regimens included folinic acid, 5-fluorouracil and oxaliplatin (FOLFOX6; 73 Cases); epirubicin, oxaliplatin and Xeloda (EOX; 12 cases); 5-fluorouracil, epidoxorubicin and mitomycin C (FEM; 9 cases); etoposide, leucovorin and 5-fluorouracil (ELF; 38 cases); mitomycin C and 5-fluorouracil (MF; 4 cases); and others (oral S-1/x, docetaxel-based and other protocols; 17 cases). All patients were followed up until January 2012. The details of the demographic and clinicopathological information were updated. The TNM stage data for the participants were obtained from the clinical and pathological diagnoses and determined according to the NCCN guidelines for GC (Version 2, 2011). The human tissue samples examined in this study were obtained from surgery and stored at room temperature after formalin-fixed and paraffinization. Correlation result displayed storage time did not affect RRM1 expression in statistical significance (*p*>0.05).

### Study Design

This was a retrospective outcome study. The sample size was estimated using nQuery Advisor 6.01 (Statistical Solutions Ltd, Saugus, MA, USA) software. Based on this calculation, a sample size of 300 participants would reach 95% study power (two-sided α = 0.05). Demographic and clinicopathological information were extracted through careful chart review. All patients were periodically followed up for survival data; patients with curative operations were also followed for recurrence-free survival. The follow-up period was calculated from the date of surgery to the date of last contact. The disease-free survival was defined as the time from the initial surgery to tumor recurrence. Metastasis or local relapse was considered evidence of tumor recurrence. Only deaths from GC were considered the endpoint of disease-specific survival. The variables assessed included birth date, gender, date of diagnosis, date and type of operation, type of chemotherapy, TNM stage, relapse/metastasis status, date of relapse/metastasis and clinical status at last follow-up.

Immunohistochemistry (IHC) was used to determine RRM1 protein expression in formalin-fixed, paraffin-embedded (FFPE) human tissue samples. To avoid systemic biases, the antibodies were validated, and the conditions of IHC were optimized using a control tissue board. To normalize the reaction conditions, all FFPE tissue samples from the ZJU set were reassembled into multiple tissue arrays. In addition, a control FFPE multiple-tissue board was included for each IHC staining. To reduce the image reader bias, an automated imaging system was employed to obtain digital images of the stained sections for subsequent quantitative analyses. Each sample was evaluated by two independent investigators in a double-blind manner.

All demographic data, clinicopathological information, and IHC results were coded and entered into a GC database. Double data entry and logic checks were used for error reduction. Microsoft Office Access® was used to create the databases. The missing cases were labeled with the appropriate “missing” code. JMP 8.0 Software (SAS Institute, Cary, NC, USA) and GraphPad Prism 5.0 (GraphPad Software, Inc, La Jolla, CA, USA) were used for the statistical analysis and survival curve plot. Multivariate logistic regression models were used to adjust for covariate effects on the odds ratio (OR). Kaplan–Meier analysis and a Cox proportional hazards model were applied for the overall survival (OS) and progression-free survival (PFS) analyses. Multivariate analyses and stratification were applied to reduce the impact of confounding effects on the estimation of hazard ratios (HRs).

### Quantitative IHC Assays

IHC was used to investigate RRM1 protein expression. The accuracy of IHC was validated by quantitative RT-PCR (qRT-PCR) on two parallel samples. Briefly, after deparaffinization, the endogenous peroxidase activity was blocked with 3% H_2_O_2_ (hydrogen peroxide). The array slides were later incubated with normal goat serum for 20 minutes, and then primary antibody was applied for 20 minutes at room temperature. After 7 minutes of H_2_O_2_ treatment, the array slides were incubated with horseradish peroxidase-labeled corresponding antibodies for 30minutes. DAB(3,3′-diaminobenzidine; 0.05 g DAB and 100 mL of 30% H_2_O_2_ in 100 mL of PBS) was applied for 5 minutes and again for 10 minutes. Each slide was then counterstained with hematoxylin (DAKO). PBS was used as a negative control.

### Antibodies

A commercially produced mouse monoclonal antibody was used against human RRM1 in this study. The RRM1 antibody production was based to our previous standard protocol [33]. Anti-hRRM1 antibody-producing hybridomas were primary screened by enzyme-linked immunosorbent assay (ELISA). Clones were chosen based on their activities on paraffin-embedded human tissues. RRM1 expression was quantified by a visual grading system based on the extent of staining. Both immunoreactivity in the nucleus and cytoplasm were evaluated. Each image was scored based on the following categories: subcellular localization (e.g., cytoplasm vs. nucleus), staining intensity (e.g., integrated optical density), and/or percentage of stained cells (e.g., total area or percentage of positive cells). Based on the intensity of the signal, RRM1 expression was classified as negative (0), weakly positive (1), positive (2) or strongly positive (3). An RRM1 score of 0 or 1 was designated RRM1-low, and a score of 2 or 3 was classified as RRM1-high. Because our data yielded consistent results between cytoplasmic RRM1 and nuclear RRM1, we considered either a high nuclear or a high cytoplasmic score as RRM1-high in the Kaplan-Meier and Cox analyses.

Antibodies against p-MEK, p-ERK, NF-κB, Ras, Sp-1 and Ki67 were obtained from Cell Signaling Technology, Santa Cruz, Invitrogen, Millipore, Abcam and BD Bioscience, respectively.

### Plasmid Construction and Transfection

The hRRM1 plasmid (pEBG-RRM1) was constructed and reported in our previous study [34]. The plasmid was transfected with X-treme GENE HP DNA Transfection Reagent (Roche Applied Science, Mannheim, Germany) according to the manufacturer’s protocol for AGS cells. Briefly, 2 µg of plasmid was mixed with 6 µL X-treme GENE HP in 200 µL serum-free medium after incubation at room temperature for 15 minutes. The mixture was added to 2×10^5^ pre-plated AGS cells in a 6-well plate. After transfection for 48 hours, the cells were harvested.

### Cell Culture and RNA Interference

The human GC cell lines AGS and NCI-N87 were obtained from American Type Culture Collection and cultured in F-12K medium (AGS) or RPMI-1640 medium (NCI-N87) (Sigma-Aldrich, St. Louis, MO, USA) supplemented with 10% fetal bovine serum (MP Biomedicals, Costa mesa, CA, USA) and 1% penicillin and streptomycin in a humidified atmosphere containing 5% CO_2_ at 37°C.

RRM1 siRNA (sc-37640) and scramble siRNA (sc-44233) were purchased from Santa Cruz Biotechnology Inc. The transfections were conducted with Lipofectamine™ RNAiMAX (Invitrogen, Carlsbad, CA, USA) according to the manufacturer’s instructions.

### *In vitro* Proliferation Assay

An *in vitro* proliferation assay was conducted according to our previous study [15]. Briefly, 0.5×10^4^ AGS and 2.0×10^4^ NCI-N87 cells were pre-plated into wells of a 16-well device compatible with a W200 real-time cell electronic sensing (RT-CES) analyzer and 16× station (ACEA Biosciences, San Diego, CA, USA). The “cell index” (normalized impedance) was calculated periodically (typically, every 30 minutes) according to the cell growth in each well. Unless otherwise indicated, four replicates were made for each siRNA treatment group. RRM1 inhibition was measured by western blotting.

### *In vitro* Invasion Assay

Matrigel invasion chambers were purchased from BD Biosciences (Franklin Lakes, NJ, USA). First, an 8-mm-porosity polycarbonate membrane was covered with 1 mL of serum-free medium containing 1×10^5^ cells per well. The plates were then incubated with a chemo-attractant (20% FBS medium) for 24 hours at 37°C in a 5% CO_2_ incubator. The medium was then removed, and non-invading cells were gently scraped off using a cell scraper. The filter was then washed twice with PBS and stained with 0.5% methylene blue for 4 hours. The cells that passed through the filter and adhered to the lower surface were counted using optical microscopy.

### Gelatin Zymography

A gelatin zymography assay was performed to investigate the influence of RRM1 on the secretion of active MMP-9. Briefly, cells were grown to 70% confluence, washed twice with 1× PBS, and incubated in serum-free medium. After 24 hours, conditioned medium was collected and concentrated with a centrifugal filter (Millipore, MA, USA) under 6000 g for 15 minutes. Concentrated samples were prepared in non-reducing sample buffer (Invitrogen, Carlsbad, CA, USA). Proteins (20 µL/lane) were separated using SDS-PAGE in gels containing 1 mg/mL gelatin (Novex 10% Gelatin Gel, Invitrogen). The gels were renatured for 1 hour at room temperature in 1× renaturing buffer (Invitrogen). Then the gels were incubated overnight at 37°C in 1× developing buffer (Invitrogen). The gel was stained with Coomassie blue. The brightness of the clear bands, where MMP9 was located and the gelatin was degraded, was analyzed using densitometry.

### Ras Activity Assay

Ras activity was measured using a Ras Activity Assay kit (Millipore, MA, USA). First, active Ras (GTP-bound Ras) was bound to the Raf-1-Ras binding domain (RBD) conjugated to agarose beads by incubating cell lysates at 4°C for 45 minutes. Then the activated Ras was released into the SDS-PAGE sample buffer after extensive washing of the agarose beads (three times) with washing buffer (25 mM HEPES (pH 7.5), 10 mM MgCl_2_, 150 mM NaCl, 1 mM EDTA, 1% Nonidet P-40, 1 mM Na_3_VO_4_, 10% glycerol, 10 µg/mL leupeptin, 10 µg/mL aprotinin and 25 mM NaF). The amount of Ras was detected with monoclonal pan-Ras antibody.

### Measurement of the dNTP Pool

This assay was conducted according to the method of Sherman and Fyfe [35]. The total reaction volume was 50 µL. The reaction mixture contained 10 mM MgCl_2_, 0.25 µM template/primer, 50 mM Tris-HCl (pH 7.5), 5 mM DTT, 1.25 µM [^3^H] dATP (for the dCTP, dGTP, and dTTP assay) or [^3^H] dTTP (for the dATP assay) and 0.2 units of Sequenase (2.0). The reaction mixture was incubated at room temperature for 20 minutes, and then the reaction was stopped on ice. After the reaction, 40-µL aliquots were removed and spotted onto circular (diameter 2.4 cm) Whatman DE81 ion-exchange papers. The papers were dried, washed (3×10 minutes) with 5% Na_2_HPO_4_, and rinsed once with distilled water and once more with 95% ethanol. Each paper was dried and deposited into a small test tube; 7 mL of Ecolume was then added to each tube. A liquid scintillation counter (Beckman Coulter, Inc. Brea, CA, USA) was used to count the tritium-labeled dNTPs. The standard samples were 0.25, 0.5, 0.75 and 1.0 pmol.

## Results

### Validation of the Specificity of the RRM1 Antibodies in the Gastric Adenocarcinoma Samples

**Figure 1:**
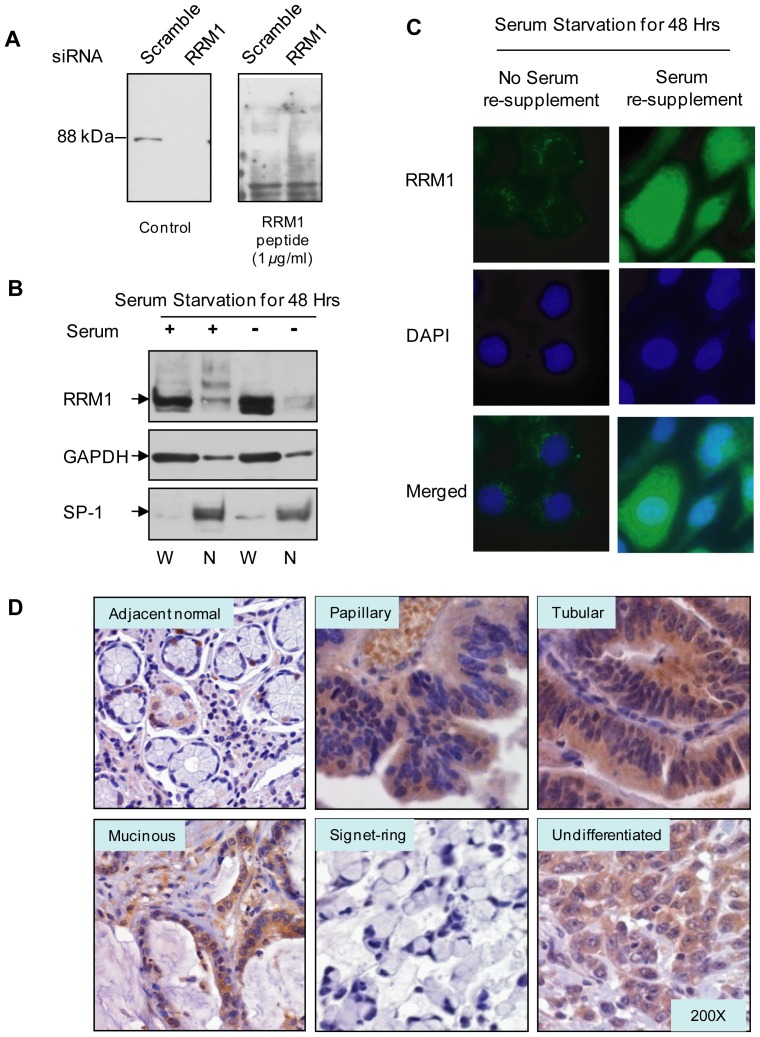
Determining RRM1 expression and localization in GC cells and tissue samples. *A*. Peptide blocking assay was conducted on the RRM1 antibodies for IHC staining, all antibodies were in 1∶1000 dilution and incubated with recombinant RRM1 peptide (1 µg/ml) at 4° overnight. Antibody #2 showed more specific and stronger signal than other antibodies (data not show), therefore we used it as the antibody for IHC staining. *B*. Nuclear fractionation was employed on AGS cells. AGS cells were starved for 96 hours and re-supplemented with normal growth medium for 12 hours. Then the cells were collected and fractionized for nuclear protein. Western blot was applied to reveal the sub-cellular localization of RRM1, GAPDH and Sp-1 were used as cytoplasmic and nuclear markers. *C*. Translocation of RRM1 from cytoplasm to nucleus was observed by Immuno-fluorescence cytochemistry. AGS cells were starved for 48 hours and re-supplemented with normal growth medium for 12 hours. Then the cells were fixed and incubated with primary and secondary antibodies. Translocation of RRM1 was observed under fluorescence microscopy. *D*. RRM1 was heterogeneously expressed among adjacent normal tissue and histological subtypes (JGCA classification V.2011) including papillary, tubular, mucinous and signet ring cell and undifferentiated adenocarcinoma.

The specificity of the RRM1 antibodies was validated by a peptide blocking assay. The antibodies were diluted (1∶1000), pre-incubated with recombinant RRM1 peptide (1 µg/mL) at 4°C overnight, and then visualized by western analysis. In Fig. 1*A*, one dominant signal of an eligible RRM1 antibody could be seen by western blot and was decreased with RRM1 knockdown in AGS cells. Meanwhile, the signal was specifically blocked by recombinant RRM1 peptide, which indicates the specificity of the RRM1 antibodies.

Because different expression patterns of RRM1 have been reported, the localization of RRM1 was further investigated. In normal cells, RRM1 was predominantly expressed in the cytoplasm (Fig. 1*B*). A fluorescence labeling assay also confirmed that RRM1 protein accumulated in the cytoplasm after 48 hours of serum starvation. However, RRM1 translocated into the nucleus in response to serum re-supplementation for 12 hours (Fig. 1*C*). Therefore, we took both cytoplasm and nuclear staining of RRM1 into consideration when interpreting the IHC staining.

RRM1 was heterogeneously expressed among the subtypes of GC tissue (Fig. 1*C*). RRM1 was preferentially expressed in cancerous tissue (47.0% RRM1-high) over the adjacent normal tissues in GC samples (25.4% RRM1-high). According to the JGCA classification [36], gastric adenocarcinoma was classified into five histological subtypes. RRM1 was highly expressed in the papillary (61.9%), tubular (59.6%) and undifferentiated adenocarcinomas (55.4%), but relatively lower in the mucinous (34.4%) and signet ring cell adenocarcinoma subtypes (14.3%).

### RRM1 Promotes Cell Growth via the Ras/Raf/MAPK Signaling Pathway in GC

**Figure 2:**
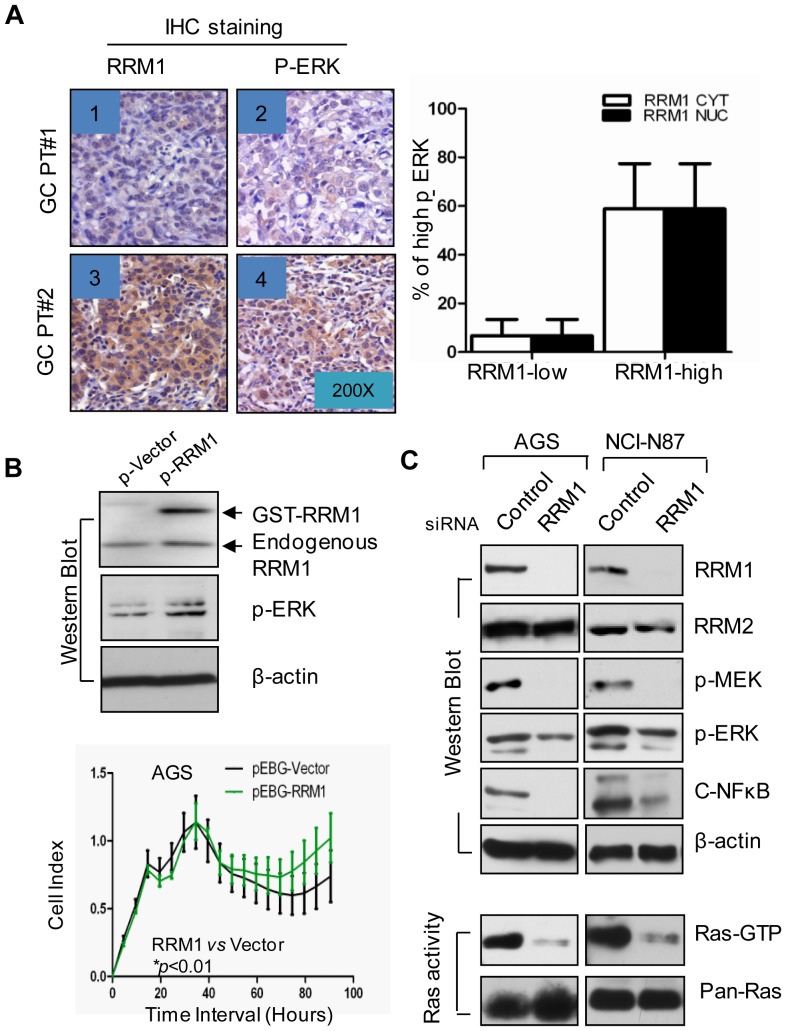
RRM1 promotes cell proliferation via Ras/Raf/MAPK pathway in GC. *A*, RRM1 overexpression was found to be accompanied with high expression of p-ERK in GC patients (*p*<0.01, n = 32), windows 1–4 show representative pictures of p-ERK and corresponsive RRM1 staining in 2 cancerous tissues from two representative patients (Pic 1–2: GC No1; Pic 3–4: GC No2). *B*, Overexpression of RRM1 (both ectopic and endogenous RRM1) through transfection of pRRM1 (pEBG-RRM1) promotes AGS cell growth; p-ERK was also up-regulated subsequently. *C*, RRM1 was down-regulated with siRNA in two GC cell lines, AGS and NCI-N87. The cells were collected and analyzed with Western blot and Ras activity kit (Millipore, MA). The Ras-Raf activity was severely decreased and so were the downstream proteins p-MEK, p-ERK and c-NFκB.

The mammalian RNR large subunit R1 (R1) suppresses malignancy in Ras transformed mouse 3T3 cells [21]. p-ERK is an important downstream component regulated by Ras/Raf signal transduction. Therefore, to study the relationship between RRM1 and GC aggressiveness, we measured p-ERK in 32 GC patients’ paraffin samples. The IHC staining indicated that RRM1 expression was concordantly associated with p-ERK level in GC samples (Fig 2*A*, *left panel*). The correlation was statistically significant (Fig 2*A*, *right panel*). Furthermore, gene transferring experiment indicated that higher RRM1 expression increased p-ERK expression in AGS cells (Fig 2*B*, *upper panel*) and promoted cell growth *in vitro* (Fig 2*B*, *lower panel*). To determine whether RRM1 regulates Ras/Raf/MAPK signaling, we down-regulated RRM1 expression by siRNA in AGS and NCI-N87 cells. Scramble siRNA was used as a negative control. Western blot analysis indicated that RRM1 was specifically reduced by the corresponding siRNA (Fig. 2*C*). Along with a reduction of RRM1, the signals for p-MEK, p-ERK and NF-κB were decreased significantly; Ras/Raf activity also dropped remarkably in both AGS and NCI-N87 cells. The above observations illustrate that RRM1 may promote GC cell proliferation by activating Ras/Raf/MAPK signaling.

### RRM1 Expression is Associated with Gastric Cancer Aggressiveness

To further explore the above findings, RRM1 protein expression was measured in the GC samples. Based on the IHC staining, 22 of 67 GCs in the COH set and 156 of 322 GCs in the ZJU set were either cytoplasmic or nuclear RRM1-high (Table 1). In the COH and ZJU sets, RRM1-high was more frequent in males than females and more likely to reach statistical significance in the ZJU set (*p*<0.01). There was more proximal GC (44.8%) in the COH set, but more distal GC (52.5%) in the ZJU set. RRM1 expression was significantly higher in the proximal GCs (*p*<0.05) in the COH set, and a similar trend was observed in the ZJU set (*p* = 0.31). Meanwhile, RRM1 was associated with the number of lymph nodes involved, tumor size, Ki67 expression, histological subtype and histological grade in the ZJU set (*p*<0.05 for each). Because of the small sample size, no statistical significance for the above factors was observed in the COH set, but similar trends could obviously be seen.

**Table 1:** Pathoclinical features of GC and IHC score of RRM1.

	COH set (n = 67)			ZJU set (n = 322)		
	No. of cases	RRM1-High^a^ N (%)	*P* value	No. of cases	RRM1-High^a^ N (%)	*P* value
Age						
<40	3	1 (33.3)		19	10 (52.6)	
40–49	7	0 (0.00)		48	18 (37.5)	
50–59	10	5 (50.0)		89	36 (40.5)	
60–69	10	3 (30.0)		83	43 (51.8)	
70–79	22	8 (36.4)		76	42 (55.3)	
>80	15	5 (33.3)	0.22	7	5 (71.4)	0.15
Gender						
Male	44	17 (38.6)		223	122 (54.7)	
Female	23	5 (21.7)	0.15	99	32 (32.3)	<0.01
Tumor Location b						
Proximal	30	16 (53.3)		61	35 (57.4)	
Body	20	4 (20.0)		73	32 (43.8)	
Distal	13	1 (7.7)	<0.01	169	79 (46.7)	0.31
Tumor Size (cm)						
<5 cm	34	10 (29.4)		125	49 (39.2)	
> = 5 cm	32	11 (34.4)	0.25	176	93 (52.8)	0.02
LN involvment^b^						
Negative	17	4 (23.5)		83	33 (39.7)	
1–2	13	3(23.1)		45	15(33.3)	
>2	36	14(38.9)	0.40	180	92 (51.1)	<0.05
Ki67 expression						
Negative	37	10 (27.0)		101	33 (32.7)	
Positve	27	11 (40.7)	0.25	192	108 (56.3)	<0.01
Distant Metastasis						
No	60	19 (31.7)		275	127 (46.2)	
Yes	6	2 (33.3)	0.93	47	27 (57.5)	0.15
Histological types						
Papillary	7	4(57.1)		21	13(61.9)	
Tubular	21	9(42.9)		47	28(59.6)	
Mucinos	2	0(0.0)		32	11(34.5)	
Signet-ring	16	4(25.0)		49	7(14.29)	
Undifferentiated	19	5(26.3)	0.41	167	92(55.1)	<0.01
Histological grade						
Low	1	0 (0.00)		24	17 (70.8)	
Moderate	19	9 (57.9)		80	41 (51.3)	
High	47	12 (23.4)	0.02	218	96 (44.0)	0.03

**NOTE:** COH: City of Hope; and ZJU: Zhejiang University. All pathoclinical information was based on diagnosis at the time of first surgery. All cases with missing information were not included in statistical analysis.

a High RRM1 includes positive and strong positive score in IHC staining.

b Tumor location:(1) Proximal includes: Cardia, GEJ, Esophagus lower, fundus; (2) Body includes: lesser curve, greater curve, stomach overlapping, body; (3) Distal includes: Gastric antrum, pylorus.

To further investigate whether RRM1 was associated with GC aggressiveness, a non-conditional multivariate logistic analysis was conducted. Here, the TNM stage was considered to be the output (stage III&IV vs. stage I&II), and the odds ratio (OR) for RRM1 (RRM1-high vs. RRM1-low) was adjusted for co-factors, including age, sex, tumor location and histological grade. The adjusted OR for RRM1 was 1.78 (95% CI 1.09–2.94) in the ZJU set. These results indicate that high RRM1 expression was associated with advanced TNM stage in GC.

### RRM1 Overexpression is Associated with Poor Survival in GC Patients

Because RRM1 is associated with advanced TNM stage of GC, Kaplan-Meier analysis and a Cox proportional hazards model were employed to determine the impact of RRM1 on the outcome of GC. In the COH set, the longest follow-up time was 228 months; 53 of 67 GC patients died from GC-related disorders, and 34 patients had a recurrence. In the ZJU set, the longest follow-up time was 179 months; a total 175 of 322 GC patients died from GC, and 87 patients had a recurrence. Results consistent with those were seen for cytoplasmic RRM1 (HR = 1.62; 95% CI 1.20–2.20) and nuclear RRM1 (HR = 1.61; 95% CI 1.19–2.18). Therefore, either high cytoplasmic or high nuclear RRM1 expression was considered in the following analysis.

**Figure 3:**
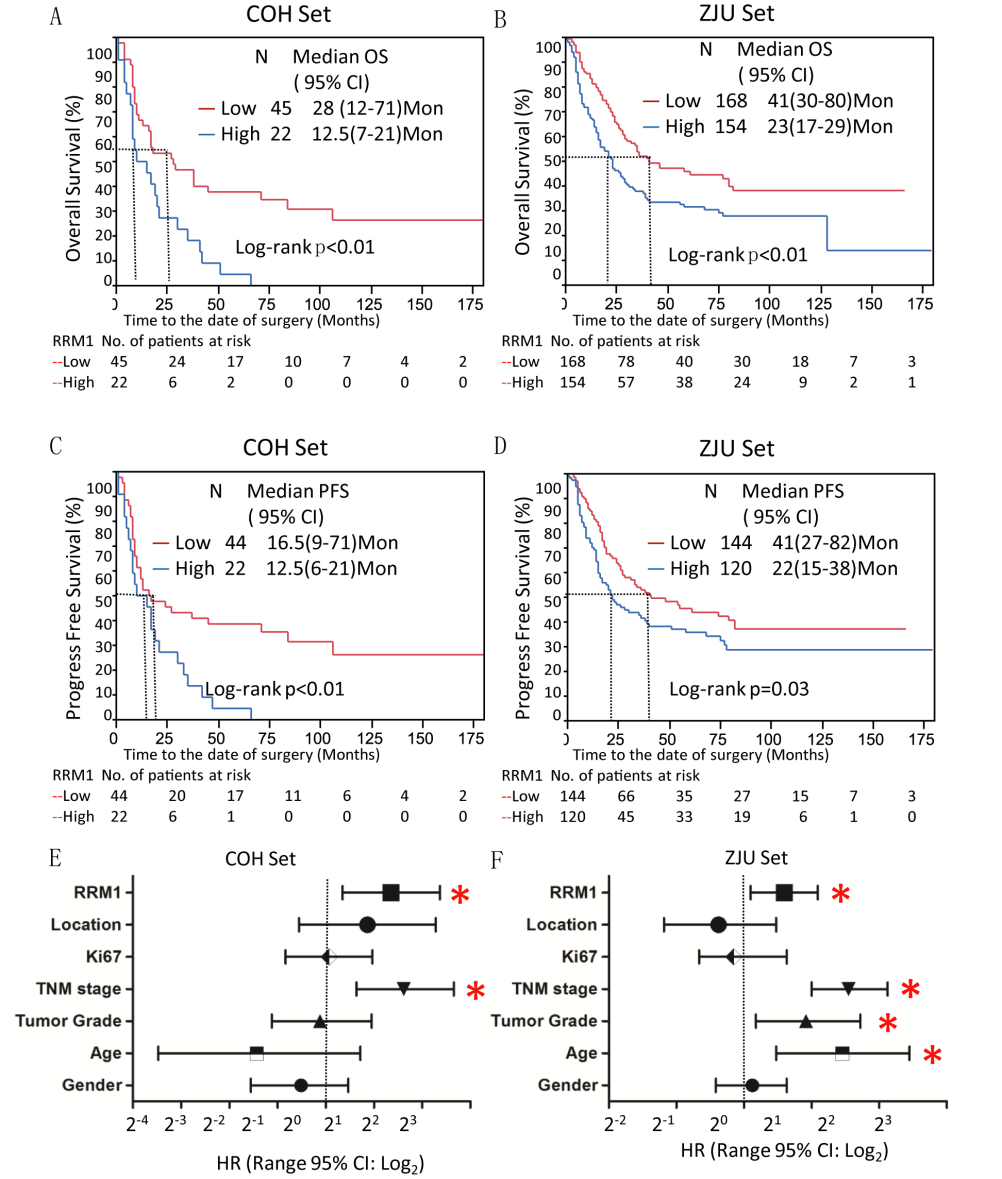
High RRM1 expression is associated with poor prognosis of GCs. *A*. The Kaplan-Meier analysis for OS was displayed as RRM1-high *vs.* RRM1-low to enhance the study power in COH set. *B*, The Kaplan-Meier analysis for OS was conducted in ZJU set. The Kaplan-Meier analysis for PFS was performed on *C* (COH set) and *D* (ZJU set). The Multivariate Cox analyses for OS of GC were shown on *E* and *F* for COH and ZJU set respectively. The hazard ratio (HR) of RRM1 was based on RRM1-high *vs.* RRM1-low; Tumor location was proximal *vs*. body *vs*. distal & Whole; Ki67 was positive *vs*. negative; Tumor stage was Stage I & II *vs*. III & IV; Tumor grade was low *vs*. moderate *vs*. high; Gender was male *vs*. female; Age was based on per unit changes. The “*” was used to indicate statistical significance (*p*<0.05).

The Kaplan-Meier analyses indicated that RRM1-high was significantly related to poor OS and PFS in the COH and ZJU sets (log rank *p*<0.01 or *p* = 0.03) (Fig. 3 *A*–*D*). The median survival time for the RRM1-low subset was 28 months in the COH set and 42 months in the ZJU set. For the RRM1-high subset, the median survival time was significantly reduced to 12.5 and 23 months in the COH and ZJU sets, respectively. Similar results were obtained in the PFS analysis. RRM1-high significantly increased the risk of GC recurrence in both sets (log rank *p*<0.01 in the COH set and *p* = 0.03 in the ZJU set).

To avoid confounder effects, a multivariate Cox analysis was conducted using the COH and ZJU sets (Fig. 3*E and 3F*). In the COH set, the factors TNM stage, tumor location, tumor grade, Ki67 level, gender and age were applied to adjust the HR. As illustrated in Figs. 3*E and 3F*, RRM1 and TNM stage were significantly associated with poor OS of GC patients in the COH and ZJU sets. The HRs for RRM1 were 2.55 (95% CI 1.27–5.15) and 1.51 (95% CI 1.07–2.13) in the COH and ZJU sets, respectively. Ki67 has been widely used as a cell proliferation biomarker for cancer, but it failed to predict the outcome of GC in the COH and ZJU sets.

### The Prognostic Performance of RRM1 in Advanced GCs

To further evaluate the prognostic performance of RRM1 in subgroups of GC, a stratification analysis was conducted. Here, we do not report the stratification results from the COH set because of the small sample size (total 67 cases). In Table 2, all of the eligible GCs were stratified by TNM stage, tumor location, histological grade, histological subtype, Ki67 expression, RRM2 expression, RRM2B expression and adjuvant chemotherapy. RRM1 more effectively predicted poor survival in patients with GC with advanced TNM stage (stage III/IV, HR = 1.55; 95% CI 1.08–2.24), higher histological grade (high, HR = 1.58; 95% CI 1.11–2.26), undifferentiated adenocarcinoma (HR = 1.73; 95% 1.14–2.67) and high proliferative potential (Ki67-positive, HR = 2.10; 95% CI 1.40–3.22). RRM1 also more effectively predicted the outcome in patients with GC located in the body of the stomach (HR = 2.10; 95% CI 1.09–4.08). We also found that the prognostic performance of RRM1 varied in the presence of different RRM2/RRM2B expression levels. RRM1 displayed more prognostic significance in the RRM2-low and RRM2B-high subgroups. Moreover, RRM1 predicted poor survival in GC patients without adjuvant chemotherapy (HR = 1.79; 95% CI 1.20–2.70) but failed to predict outcomes in patients who received adjuvant chemotherapy. Taken together, the above analyses suggest that RRM1 specifically predicts the outcome of patients with advanced GCs with poor differentiation and high proliferation.

**Table 2:** Stratification analysis for expression of RRM1 at primary cancer and survival of GC patients in ZJU set.

	N	HR (95% CI)	Adjusted HR(95% CI)
TNM stage			
Stage I & II	127	0.93(0.47–1.76)	0.92 (0.47–1.76)
Stage III & IV	189	1.63(1.14–2.34)*	1.55 (1.08–2.24)*
Tumor location			
Proximal	61	1.69(0.85–3.58)	1.66 (0.83–3.57)
Body	73	1.96(1.07–3.63)*	2.10 (1.09–4.08)*
Distal	169	1.34(0.86–2.11)	1.32 (0.84–2.08)
Histological Grade			
Low	24	1.88(0.54–8.62)	1.18(0.19–10.3)
Moderate	80	2.45(1.24–5.17)*	2.20 (1.11–4.57)
High	218	1.79(1.22–2.62)*	1.58 (1.11–2.26)*
Histological Subtype			
Papillary+Tubular	68	1.65(0.75–3.86)	1.82(0.80–4.47)
Mucinos+Signet-Ring	81	2.32(1.18–4.32)*	1.69(0.81–3.38)
Undifferentiated	167	1.59(1.06–2.43)*	1.73(1.14–2.67)*
Ki67			
Negative	101	1.30(0.74–2.21)	1.18 (0.66–2.09)
Positive	192	2.09(1.39–3.20)*	2.10 (1.40–3.22)*
RRM2			
Low	150	1.96(1.26–3.07)*	1.84(1.17–2.88)*
High	148	1.30(0.82–2.1)	1.37(0.86–2.23)
RRM2B			
Low	142	1.40(0.82–2.28)	1.41(0.83–2.32)
High	165	1.82(1.14–3.01)*	1.89(1.18–3.16)*
Chemotherapy			
No	169	1.53(1.12–2.11)*	1.79 (1.20–2.70)*
Yes	153	2.69(0.89–9.84)	1.38 (0.88–2.18)

**NOTE:** Multivariate COX proportional hazard analysis was conducted to evaluate HR of RRM1. The adjusted HR was adjusted by sex and age at diagnosis. The TNM stage was based on tumor invasion, lymph node involvement, and distance organ metastasis. HR of RRM1 was based on high expression *versus* low expression.

* Statistics significant on COX analysis, *p*<0.05.

### Inhibition of RRM1 Reduces the Proliferative and Invasive Abilities of GC Cells

**Figure 4:**
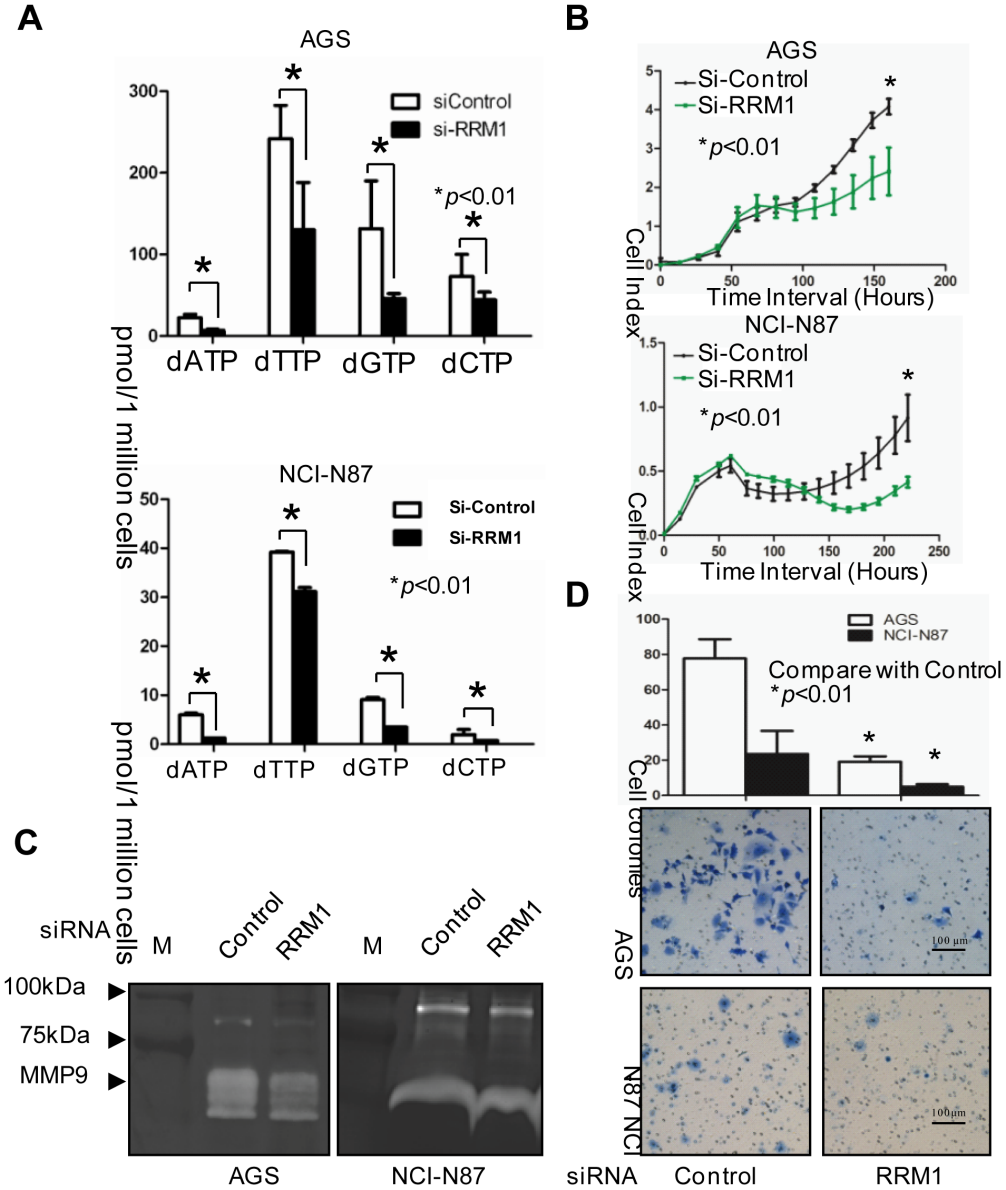
Inhibition of RRM1 significantly reduced proliferative and invasive abilities of GC cells. *A*, dNTP pools level were significantly reduced after RRM1 depletion in AGS and NCI-N87 cells. *B*, Real-time cell growth assay was performed in GC cell lines AGS and NCI-N87. RRM1 knockdown cells showed distinctly impaired growth compare to control cells. *C,* RRM1 inhibition significantly reduced the secretion of active MMP-9. Gelatin zymography was conducted in AGS and NCI-N87 cells. Briefly, Cells post-siRNA transfection (48 hours) were counted and seeded in a 10 cm dish (1 million/dish), the supernatant was collected after 24 hours of starvation with serum free medium. After centrifugal filtration, the concentrated supernatant was analyzed for active MMP-9 level with gelatin gel by SDS-PAGE. Eventually, we found the secretion of active MMP-9 was obviously lower in RRM1 knockdown cells; *D*, *In vitro* invasion chamber assay was employed to show the impact of RRM1 on cell invasion. An significant reduction (*p*<0.01) in invasive ability was observed in RRM1 knockdown cells.

The above findings show the prognostic value and possible mechanism of RRM1 in GC. RRM1 is considered a therapeutic target for cancer treatment since it is involved in providing dNTPs for DNA synthesis [10]. Here, we further demonstrate that down-regulating RRM1 expression by siRNA reduced proliferation and invasion in GC cells. In Fig. 4*A*, a significant reduction of the dNTP pool was observed in the RRM1-knockdown GC cells. Subsequently, with the deficiency of dNTPs for DNA synthesis, distinct growth retardation could be seen in the RRM1-depleted cells compared to the control siRNA-treated cells (Fig. 4*B*). Moreover, we conducted gelatin zymography and invasion chamber assays to examine the role of RRM1 in the invasiveness of GC cells. After treatment with anti-RRM1 siRNA, a reduction of active MMP9 was seen (Fig. 4*C*). Meanwhile, the invasive potential decreased (*p*<0.01) (Fig. 4*D*). Thus, these findings suggest that inhibition of RRM1 significantly reduces the proliferation and invasion of GC cells.

## Discussion

In this study, we examined the protein expression of RRM1 to evaluate its potential value as a prognostic marker in GCs. RRM1 expression was significantly higher in cancerous tissue and heterogeneously expressed in the histological subtypes of gastric adenocarcinoma. RRM1 expression was also significantly associated with number of lymph nodes involved, tumor size, Ki67 expression, histological subtype and histological grade. RRM1 also significantly predicted a poor prognosis in two independent GC populations (COH and ZJU sets) with different racial and socio-economic backgrounds (Fig. 3), suggesting that RRM1 might serve as a potential biomarker for predicting poor survival in GC patients. Moreover, stratification analysis illustrated that RRM1 was more effective in predicting poor survival in GC patients with advanced TNM stage, poor differentiation and high proliferative potential. Here, *in vitro* experiments demonstrated that inhibition of RRM1 by siRNA significantly reduced the dNTP pool, Ras/Raf and MMP-9 activities and p-MEK, p-ERK and NF-κB levels, which resulted in growth retardation and reduced invasion in AGS and NCI-N87 cells. The above findings suggest that RRM1 might be not only a prognostic biomarker but also a potential therapeutic target in GCs.

Previous studies have demonstrated the association between RRM1 protein expression and cancer outcome, but their conclusions vary [23]–[25], [27]–[29], [37]. The different results obtained by these studies might have been caused by multiple factors: 1) different types of cancer; 2) different cancer treatments; and 3) participant heterogeneity (stages and races). First, analysis of published microarray data from ONCOMINE indicated that regulation of RRM1 mRNA was divergent in different caner types. The roles of RRM1 in different cancers are still largely unknown. RRM2 and RRM2B, the two human RNR small subunits, can bind to RRM1 to form a holoenzyme. Because RRM2 and RRM2B are always regulated oppositely throughout the cell cycle, the prognostic performance of RRM1 varies with the RRM2/RRM2B expression levels. RRM2B is commonly detected at G1 phase, but RRM2 is an S phase-specific protein [38]. RRM1 is always co-expressed with RRM2B, rather than RRM2, in resting cells. Activation of the RNR enzyme might result in an expanding and unbalanced dNTP pool [39]. RNR can enhance cancer cell proliferation, invasion and genomic instability, which potentially causes poor outcome in cancer patients. This mechanism could partially explain the poor GC survival in the RRM2B-high and RRM2-low subgroups (Table 2). Also, RRM1 was correlated with expression of the DNA repair protein ERCC1 and was associated with better outcome of early-stage non-small-cell lung cancer [23]. It also could suppress malignancy by inducing *PTEN*, a tumor suppressor gene [26]. PTEN suppress the phosphoinositide 3-kinase (PI3K)-AKT-mammalian target of rapamycin (mTOR) pathway through its lipid phosphatase activity [40]–[41]. *PTEN* loss and RAS/MAPK activation could cooperate to promote epithelial-to-mesenchymal transition (EMT) and metastasis initiated from prostate cancer stem/progenitor cells [42]. Therefore, RRM1 might potentially lead to better outcomes of some cancer patients. Different treatments also could alter the roles of RRM1 in cancers. Expanding the dCTP pool by RRM1 overexpression could block gemcitabine incorporation into DNA through competitive binding [30]. This mechanism is one way RRM1 causes gemcitabine drug resistance. siRNA-mediated down-regulation of RRM1 sensitizes lung cancer cells to gemcitabine treatment *in vivo* and *in vitro* [43]. Gemcitabine has only been used to treat GCs in a few cases [44]. Currently, chemotherapy for GCs is based on a 5-fluorouracil protocol. RRM1 overexpression has been correlated with 5-fluorouracil resistance in pancreatic cancer [45]. In table 2, highly expression of RRM1 impact the poor outcome of GC patients with 5-fluorouracil based chemotherapy, but not significantly. It revealed that the role of RRM1 in drug resistance of GC needs to be further explored. Due to these potential confounding factors, TNM stage, tumor location, tumor grade, tumor size, Ki67 level and chemotherapy were taken into consideration in the stratification analysis. Some variances were observed in the different subgroups (Table 2), but overall, the data indicate that RRM1 predicts poor prognosis in GC.

Different antibodies used for detecting RRM1 protein expression and localization also could potentially lead to different conclusions. Certain antibodies, such as R1AS6, recognize mainly nuclear RRM1 [23], [46], but others visualize the cytoplasm-dominant IHC pattern, such as the RRM1 polyclonal antibody from the Protein Tech group [29], [47] and Accurate Chemicals AD203 [21]. To avoid this type of bias, we developed our own monoclonal antibodies. The sensitivity of the antibodies for IHC was optimized, and the specificity was verified by a peptide blocking test (Fig. 1*A*). Therefore, we believe the RRM1 antibodies used in this study were reliable. We observed that RRM1 was dominantly located in the cytoplasm during serum starvation and translocated to the nucleus under serum re-supplementation (Fig. 1*B and 1C*). In human GC tissue samples, RRM1 was seen heterogeneously in the cytoplasm and nucleus by IHC staining (Fig. 1*D*). The clinical relevance of cytoplasmic and nuclear RRM1 was evaluated independently. Consistent results were obtained from cytoplasmic and nuclear RRM1 scoring. These results suggest that both cytoplasmic and nuclear RRM1 are significantly associated with advanced TNM stage and lead to poor outcomes in GC patients.

The mammalian RNR subunits R1 and R2 play opposing roles in malignancy suppression/progression through the Ras/Raf/MAPK signaling pathway in Ras-transformed 3T3 cells [21]–[22]. And there are also some studies explain why RRM1 and RRM2 play opposite roles in cancer outcomes [17]–[18], [27], [48]. Here, the relationship between RRM1 and the malignancy of GCs was further examined in cultured cells and tissue samples. In human subjects, we detected a positive association between RRM1 and p-ERK in GC samples (Fig. 2*A*). The *in vitro* data reveal that high RRM1 expression significantly increased p-ERK and cell proliferation (Fig. 2*B*). To further explore the role of RRM1, we down-regulated RRM1 using siRNA in AGS and NCI-N87 GC cells. With RRM1 knocked-down, Ras/Raf activation was suppressed, and p-MEK and p-ERK also decreased significantly (Fig. 2*C*). The reduction of the dNTP pool and growth retardation could be seen in AGS and NCI-N87 cells after RRM1 siRNA treatment (Fig. 4*A and 4B*), which is consistent with the fact that the Ras/Raf/MAPK signaling pathway is related to cancer cell growth and metastasis [49]. The gelatin zymography assay demonstrated that MMP activity decreased upon down-regulation of RRM1 (Fig. 4*C*). Inhibiting RRM1 expression also decreased the invasion ability of GC cells (Fig. 4*D*). Obviously, this result is not consistent with previous findings using NIH 3T3 cells [21]. The conflicting results might be caused by using different cell types and species. Regardless, this result was compatible with our findings in human subjects.

There were some limitations to the current study. First, certain biases, such as selection bias, observer bias, measurement bias and confounders could not be completely avoided in this retrospective study. Nevertheless, these limitations have been taken into consideration. To make our conclusion more reliable, we collected two sets of GC patients with different races and socio-economic backgrounds to validate our findings. The specificity of the antibodies used was confirmed and optimized before being applied to the study. Double-blinded IHC scoring was used to reduce the observation and measurement bias. The multivariate and stratification analyses were conducted to reduce the confounder effects as much as possible. Another limitation was that we overexpressed RRM1 in AGS cells, but not in NCI-N87 cells. Further investigation is necessary to delineate the mechanism by which RRM1 promotes GC aggressiveness.

**Figure 5:**
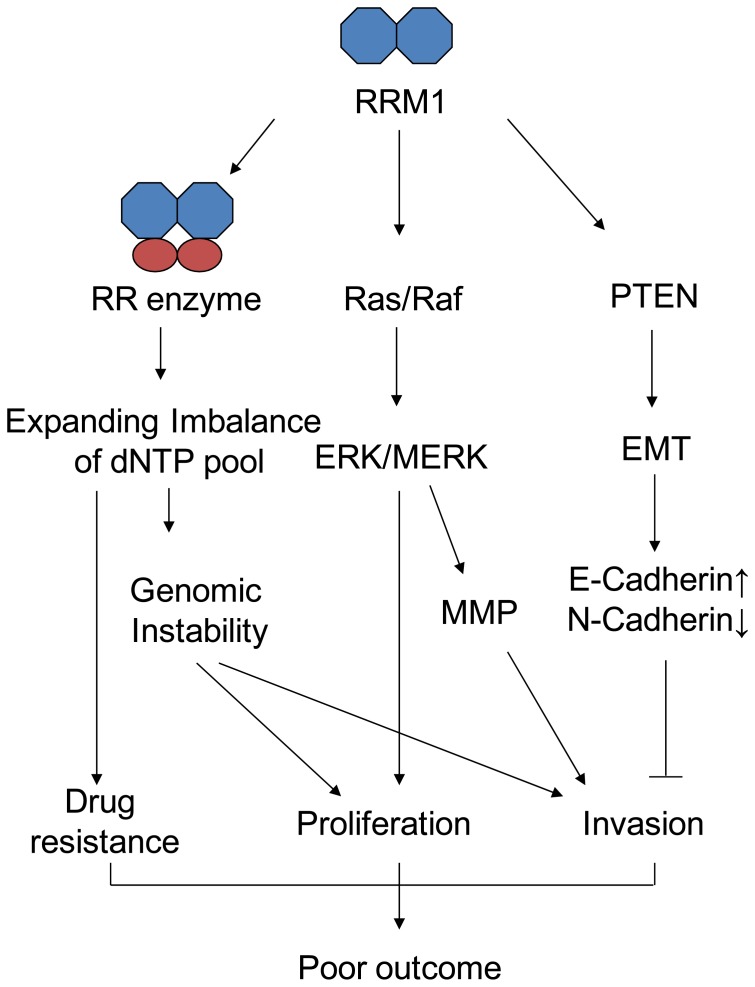
A schematic summary of RRM1’s role in cancer development and treatment. RRM1 regulates the proliferation and invasion through dNTP pool level, Ras/Raf/MAPK signal pathway. Also, it could induce the expression of PTEN and set impact on the EMT (Epithelial-to-Mesenchymal Transition) [see ref 40].

In summary, we demonstrated that RRM1 overexpression was associated with poor prognosis in GC patients, especially advanced-stage GC. Also, RRM1 inhibition reduced proliferation and invasion in GC cells via the Ras/Raf/MAPK pathway (Fig. 5). Therefore, RRM1 may be a potential prognostic and therapeutic biomarker in GC patients.
